# A new substance to relax polychaete worms (Annelida) prior to morphological study

**DOI:** 10.3897/zookeys.594.8061

**Published:** 2016-05-30

**Authors:** Alieh Bonyadi-Naeini, Hassan Rahimian, Christopher J. Glasby

**Affiliations:** 1 Dept. of Biology, Faculty of Science, Razi University, Baghe- e- Abrisham 67149 Kermanshah, Iran; 2 Faculty of Biology, College of Science, University of Tehran, Tehran, Iran, P.O.Box 14155–6455; 3 Museum and Art Gallery of the Northern Territory, P.O. Box 4646, Darwin, Northern Territory 0801, Australia

**Keywords:** Carvacrol, narcotize, Polychaeta, specimen preparation

## Abstract

A variety of chemical substances have been used to relax and/or immobilize polychaete worms, and other invertebrates, prior to specimen preparation for morphological examination. To solve difficulties encountered during the study of nereidid polychaetes (Annelida: Phyllodocida), an experiment was designed and carried out to investigate a new relaxing agent to immobilize nereidid specimens and stimulate pharynx eversion. The new substance, Dentol® (Khoraman laboratory, Iran), a dental anesthetic and antiseptic medicine containing 10% Carvacrol as the effective ingredient, was used for the first time and compared with other substances that have been used traditionally in polychaete studies. Crosstab analysis showed significant differences between different treatment groups, with Dentol® providing much better results for all considered criteria.

## Introduction

In order to properly examine nereidid polychaetes, and other Phyllodocida (Annelida), they first should be relaxed, to expose their eversible pharyngeal organ, and in the case of nereidids their pharyngeal armature (paragnaths and/or papillae) and jaws. Anesthetization facilitates pharyngeal organ eversion (Oliveira et al. 2010). Various anesthetics, including Magnesium Chloride (Fauchald 1977; Rouse and Pleijel 2001), Menthol crystals (Coutinho and Santos 2014; Glasby and Hsieh 2006; Russell 1963), MS222 (Tricaine Methane Sulphonate) (Clark 1961; Fauchald 1977), Phenoxyethanol (Hsieh and Li 1998), Ethyl Alcohol (Russell 1963), and various techniques such as cooling the worms (Costa-Paiva et al. 2007), have been applied to immobilize and/or relax polychaetes. The benefits of anesthetization include reducing the risk of morphological damage (e.g., loss of body segments and parapodial appendages) during fixation, and slowing the specimen down to enable morphological examination of live specimens and photography. Disadvantages include the additional time required for sample preparation (time of relaxation varies with taxa from minutes to hours), and the possibility of distortion of morphological features. For example, inadequate fixation methods or using different anesthetics have resulted in erroneous taxonomical observations of *Laeonereis* (Oliveira et al. 2010) and sabellid polychaetes (Costa-Paiva et al. 2007). A suitable anesthetic substance should be safe, environmentally friendly, readily available, easy to use, economically feasible, effective, efficient, and without any morphological modifications resulting from over-relaxation, for example (Costa-Paiva et al. 2007, Williams and Van Syoc 2007).

During a survey of nereidids of the Persian Gulf, we encountered some difficulties concerning anesthetic substances, mainly poor results for pharyngeal eversion and a long elapsed-time to relaxation. Most previous studies concerning anaesthetization methods are about the maintenance of living animals for transportation rather than preparation for morphological studies (Oliveira et al. 2010). Consequently, in search for better results, we designed a study to find a more effective, efficient and readily available anesthetic agent to relax specimens and stimulate nereidids to evert their pharynx. In this study we compare three conventional substances previously used in other studies (Beesley et al. 2000; Fauchald 1977; Russell 1963) with a new substance, Dentol®, a dental anesthetic and antiseptic medicine containing 10% pure Carvacrol; Although the availability of Dentol® outside of Iran has not been determined, Carvacrol at least is present in different concentrations in oil of oregano, oil of thyme, oil obtained from pepperwort, and wild bergamot and many other plant extracts, available worldwide (see Discussion).

## Material and methods

Nereidid worms were collected from the intertidal zone of two islands, Qeshm (26°58'17"N, 56°15'32"E) and Hengam (27°03'01"N, 56°29'58"E), west of the Strait of Hormuz, the Persian Gulf.

A total of 60 specimens of different species were randomly separated into four equal groups, each containing 15 specimens. The first group was administered with 8% MgCl_2_ in seawater, the second group with 8% MgSO_4_ in seawater, the third group with 8% Menthol crystals in sea water, and the fourth group with 8% Dentol® (10% pure Carvacrol, or cymophenol, C6H3CH3(OH)(C3H7), a monoterpenoid phenol) in seawater. Specimens in each group were exposed to the relevant substance and the time to immobilization recorded. After 30 minutes, specimens in each treatment group were fixed, separately, in 5% formaldehyde diluted in seawater, for 24h. After fixation, specimens were washed in tap water and transferred into 70% ethyl alcohol for storage. Prepared specimens were studied under a stereo microscope, to compare how the different treatments affected morphology.

**Figure 1.**
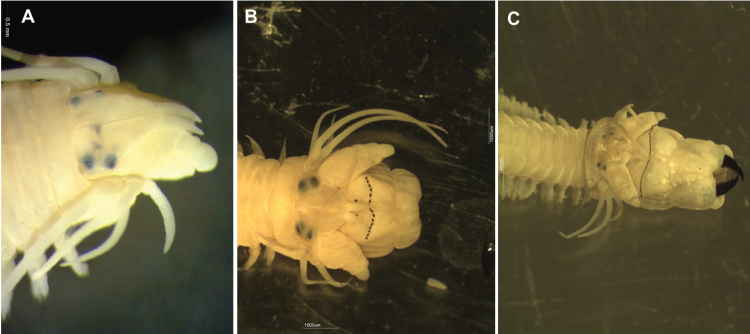
Categorization of nereidid worms based on the amount of proboscis eversion, after anaesthetic applications; not-everted (**a**), partially-everted (**b**), and fully-everted (**c**).

In all groups, based on the amount of proboscis eversion, specimens were classified into three categories, not-everted (Figure 1a), partially-everted (Figure 1b), and fully-everted (Figure 1c). Specimens in each category were counted, their length and width were measured, to the nearest millimeter, and then weighed individually to the nearest milligram. Measurements were entered into SPSS (ver. 22) and statistical analyses including crosstab, chi-square, t-test and ANOVA were carried out.

## Results

Mean and standard deviation for length, width, and weight of specimens for each treatment group were measured to the nearest millimeter. ANOVA analysis showed no significant difference between the four treatment groups for length (F=2.011, df=3, P=0.052), width (F=0.892, df=3, P=0.451), and weight (F=1.686, df=3, P=0.180) (Table 1).

**Table 1.** Descriptive statistics, ANOVA and crosstab analysis for the effect of different substances on pharynx eversion in the four treatment groups.

Treatments	Number of specimens	Length (mm)		Width (mm)		Weight (gr)		ANOVA			Everted No. (percentage)			Crosstab
		M	SD	M	SD	M	SD	length	width	weight	Not-everted	Partly-everted	Fully- Everted	
**Dentol**	15	61.21	15.87	3.69	0.58	0.125	0.058	F=2.011, df=3, P=0.052	F=0.892, df=3, P=0.451	F=1.686, df=3, P=0.180	0	0	15 (100%)	χ^2^=78.462, df=6, *P*<0.001
**Menthol**	15	73.00	16.69	3.43	0.45	0.164	0.054				15 (100%)	0	0	
**Mg Cl_2_**	15	77.19	16.75	3.55	0.44	0.153	0.045				15 (100%)	0	0	
**Mg SO_4_**	15	60.71	15.15	3.71	0.63	0.165	0.062				9 (60%)	6 (40%)	0	

Examining the number of specimens with an everted pharynx for each treatment showed that Dentol® treated worms had 15 specimens (100%) with a fully everted pharynx. By comparison, Menthol and MgCl_2_ treatments had no specimens with an everted pharynx after a 30 minute exposure period. In the MgSO_4_ treatment group nine specimens (60%) had an uneverted pharynx, while six specimens (40%) had a partially-everted pharynx. Crosstab analysis showed significant differences between different treatment groups in the number of everted pharynges (χ2=78.462, df=6, P<0.001) (Table 1). Further, specimens of the Dentol® treated group became motionless faster than those with other treatments (less than one minute compared to more than one minute).

## Discussion

Most polychaetes have an eversible pharynx, the anterior part of digestive tract (Fauchald 1977), which has taxonomically useful features on its surface. Members of Nereididae have small horny teeth (paragnaths) and/or papillae whose various shapes, patterns and numbers can be used in generic and species identification (Bakken et al. 2009; Fauchald 1977; Uschakov 1955). The problem, however, is that the pharynx is not readily everted and its taxonomically-useful characteristics are often not visible in preserved specimens. In these cases dissection with iridology scissors is the only means to reveal and observe the pharyngeal characters; however, specimens less than about 1 mm are impossible to dissect even with the finest scissors. As a result, different substances have been used to facilitate pharynx eversion. The most frequently used substance, mentioned in scientific papers, is 7–8% Magnesium Chloride (MgCl_2_) in seawater (MgCl_2_ .6H_2_O) (Bonyadi and Rahimian 2009; Darbyshire 2014; Fauchald 1977; Williams and Van Syoc 2007), although Rouse and Fauchald (1997) recommend dilution in fresh water. Time to relaxation is at least 30 minutes on small-sized worms. Magnesium chloride works because Magnesium ions can relax muscles through direct action on cell membranes; Mg^2+^ ions block certain types of Calcium channels, which conduct a positively charged Calcium ion into neurons. An excess of Magnesium, causes more channels to be blocked resulting in nerve cells having less activity (Slutsky et al. 2010), hence muscle relaxation and pharynx eversion. Menthol crystals are a slower acting anesthetic, needing about 12 hours (Wilson et al. 2003) to take the desired effect. Menthol works by blocking voltage-sensitive sodium channels, hence reducing neural activity that may stimulate muscles (Haeseler et al. 2002). MS-222, Tricaine methanesulfonate (a chemical commonly used for anesthesia, sedation, or euthanasia of fish) (Wayson 1976), has also been used to relax polychaetes prior to examination (Fauchald 1977; Fidalgo e Costa 1999). MS-222 can simultaneously block pain sensation, paralyze the animal by muscle relaxation and exert a general anesthesia by blocking Na^+^-conductance of cellular elements comprising the neuromuscular system (brain and muscles) (Ramlochansingh et al. 2014). A 0.05 percent solution of MS-222 in sea-water completely anesthetizes medium-sized worms within 5–7 minutes (Clark 1961). Other substances including Phenoxyethanol (McKay and Hartzband 1970), Ethyl Alcohol 70% (Russell 1963), and Clove oil (Hutchings and Glasby 1999; Rouse and Fauchald 1997) have also been used to evert a polychaete’s pharynx, but much less frequently. Finally, fast acting anesthetics are preferable in general, in order that the integrity and quality of the specimens is maintained, particularly in tropical areas where delays can result in specimen degradation.

Dentol®, contains 10% pure Carvacrol, an essential oil, or terpene, present in various aromatic plants. Carvacrol is the main oil constituent (86%) in *Satureja khuzistanica* Jamzad (Marzeh khuzestani in Persian, family Lamiaceae). *Satureja khuzistanica* is an endemic plant widely distributed in the southern parts of Iran (Jamzad 1994) and has been traditionally used as an analgesic and antiseptic agent (Farsam et al. 2004). The effective agent of this plant, Carvacrol, has anti-inflammatory, antinociceptive, antidiabetic, and antioxidant properties (Baser 2008; Clark 1961; Rahman 2013). Moreover, the pharmacological inhibition of smooth muscular activity has been attributed to the prostaglandin inhibition effects of Carvacrol (Kintzios 2003).

Carvacrol is also present in two other well-known, herbaceous plant derived oils, although in lesser amounts: Oregano Oil (*Oreganum compactum*, 50%), and Clove Oil (*Eugenia caryophyllata*, <1%) (Charai et al. 1996; Chaieb et al. 2007). Since Carvacrol is present in all three plant extracts, a comparison of their anesthetic effects on polychaetes would be useful, and may assist in determining whether Carvacrol itself, or one of the other aromatic oils, or perhaps a synergistic combination of oils, is responsible for the anesthetic effect. One possible disadvantage of the use of essential oils for anesthetization is that specimens may be coated with an oily film that does not dissolve readily in ethanol preservative and so could be problematic especially in molecular studies; this possibility, however, needs to be investigated.

Results of the present experiment showed significant differences between different substances used to narcotize/relax polychaete worms. In examining polychaete worms it is important not only to immobilize the specimen but also to have its pharynx everted to be able to observe its taxonomically important characteristics.

Like Nereididae, other members of Phyllodocida have a symmetrical axial pharynx with a strong muscular region that is often protractible (Dales 1962; Rouse and Fauchald 1997). Therefore Dentol® may be an effective anesthetizing agent for other Phyllodocida families, such as Hesionidae, Pilargidae, Syllidae, etc., although the precise details of their response would need to be examined and compared to that in Nereididae.

The mechanism of pharynx eversion appears to be the same in all groups of nereidids though there are differences in detail (Dales 1962), and knowing that samples tested included different species, it is likely that some of the variation between treatments could be the result of variability between species.

One ‘manual’ way to achieve pharynx eversion is to anesthetize the worm and then encourage them to evert the pharynx by stimulating, pressing or otherwise forcing it to eject the pharynx. That, however, is not an easy task requiring considerable microscopic manipulation skills, and is time consuming particularly if many specimens are to be treated. Using Dentol® has the advantages of both immobilizing the specimen (for example for photography) and everting the pharynx, both in a short time. This substance, and/or its active ingredient Carvacrol, is effective, safe, reasonably cheap, and usually easy to obtain in pharmacies in many countries around the world.
